# Effect of Complex Strengthening on the Continuous Cooling Transformation Behavior of High-Strength Rebar

**DOI:** 10.3390/ma15248940

**Published:** 2022-12-14

**Authors:** Jingtian You, Zhiying Li, Jie Wang, Changrong Li, Zeyun Zeng, Shiwang Li, Sheng Huang

**Affiliations:** 1 College of Materials and Metallurgy, Guizhou University, Guiyang 550025, China; 2 Guizhou Provincial Key Laboratory of Metallurgical Engineering and Process Energy Saving, Guiyang 550025, China; 3 Shougang Shuicheng Iron and Steel (Group) Co., Ltd., Liupanshui 553000, China

**Keywords:** high-strength rebar, phase transformation characteristics, cooling rate, bainite, (Nb, Ti, V)C

## Abstract

The effects of niobium and composite strengthening on the phase transformation characteristics and precipitation behavior of continuous cooling transformation of high-strength rebar during thermal deformation and subsequent cooling were investigated. The results show that when the cooling rate was within 0.3–5 °C/s, ferrite transformation and pearlite transformation occurred in the experimental steels. The Nb content increased to 0.062 wt.%, and the starting temperature of the ferrite transformation decreased. Meanwhile, the ferrite phase transformation zone gradually expanded, and the pearlite phase transformation zone gradually narrowed with the increase in the cooling rate. When the cooling rate was 1 °C/s, bainite transformation began to occur, and the amount of transformation increased with the increase in the cooling rate. It was found that the main precipitates in the experimental steels were (Nb, Ti, V)C, with an average particle size of about 10–50 nm. When the Nb content was increased to 0.062 wt.% and the cooling rate was increased to 5 °C/s, the ferrite grain size was reduced from 19.5 to 7.5 μm, and the particle size of the precipitate (Nb, Ti, V)C could be effectively reduced. The strength of the steel was significantly improved, but the elongation of the steel was reduced. However, the comprehensive mechanical properties of 0.062 wt.% Nb experimental steel was significantly improved at a cooling rate of 5 °C/s.

## 1. Introduction

High-Strength steel bars are widely used in large construction projects such as bridges and tall buildings [1,2]. The new global situation of carbon peaking and carbon neutrality and the revision of a new national standard for hot-rolled steel bars in China (GB/T 1499.2–2018) have put forward new demand for construction steel bars. Therefore, it is important to improve the strength of steel for reinforced concrete and explore the new process of microalloyed High-Strength reinforcing steel. In microalloyed hot-rolled steel, by optimizing the composition of trace alloying elements such as Ti, V, and Nb, combined with the controlled rolling and cooling process, the organization of microalloyed hot-rolled steel can be effectively improved, which ensures excellent strength and toughness [3,4,5,6]. The improvement in properties is mainly due to grain refinement and precipitation strengthening of precipitates (carbides, nitrides or carbonitrides) formed by microalloy elements at different process stages [7,8,9,10]. Nb is extremely easy to combine with C and N. Nb precipitates effectively refine austenite grains by hindering recovery and recrystallization through solute dragging and precipitate pegging during thermal deformation [11,12,13,14,15].

The role of Nb in ferritic steels has been extensively studied. Xin et al. [16] showed that microalloyed steels with 0.093% Nb could promote the precipitation of a large amount of carbide and thus improve high-temperature strength; Nb could effectively refine the grain during rolling and improve room-temperature strength and low-temperature toughness. Jung et al. [17] quantitatively investigated the dissolution behavior of complex carbonitrides in Nb–Ti–V microalloyed steels. It was found that the optimum solution treatment temperature of Nb–Ti–V microalloyed steel was 1200 °C. At the solution treatment temperature above 1200 °C, irregularly shaped precipitates rich in Nb and C disappeared, and cuboid precipitates rich in Ti and N still existed. Hong et al. [18] observed dendritic Nb-rich (Ti, Nb)(C, N) in as-cast slab steel, which has a thermodynamically stable chemical composition below 1000 °C. Dong et al. [19] studied the phase transformation behavior of Nb–V–Ti microalloyed ultra-high strength steel during continuous cooling and found that the starting temperature of ferrite transformation (A_r3_) decreased with the increase of cooling rate, which was related to the precipitation of carbides. Yang et al. [20] characterized and analyzed the evolution and effect of Nb/Ti composite inclusions, where dislocation motion was suppressed due to the pinning effect of reprecipitated particles. In addition, the addition of 0.045 wt.% Nb changed the size, morphology, and composition of nano-scale precipitates.

In addition to the refinement of microstructure and the formation of precipitates, the effect of Nb addition on bainite transformation is also the main reason to improve the strength of steel [21]. Huang et al. [10] investigated the effect of Nb on the microstructure and properties of Ti–Mo microalloyed high strength steel and found that Nb accelerated the precipitation of precipitates in the steel, which is beneficial to obtain finer ferrite grains and inhibits bainite transformation. Studies have also shown that [22,23], with the increase in Nb content, the bainite starting temperature continued to decline, delaying the transformation of bainite. However, Rees et al. [24] and Wang et al. [25] studied the effects of solid solution niobium in austenite on the kinetics of bainite transformation during cooling using a dilatometer, scanning electron microscope and transmission electron microscope and pointed out that the increase in solid solution niobium promoted bainite transformation. In addition, Cai et al. [26] studied the effect of Nb on the transformation of ferrite and bainite in microalloyed steel and concluded that the phase transformation kinetics of ferrite and bainite showed a similar trend with increasing Nb content, that is, Nb first inhibited and then accelerated. Compared with Nb-free steel, the addition of 0.046 wt.% Nb significantly accelerated the ferrite transformation kinetics but inhibited the bainite transformation.

In summary, there are some controversies about the effect of Nb content on the microstructure and properties of microalloyed steel, especially the effect on bainite transformation in steel. In addition, the combined effects of Nb and different continuous cooling rates on the phase transition characteristics and the morphology and size of Nb/Ti/V composite precipitates have not been extensively studied. Therefore, in this study, two kinds of microalloyed steels with different niobium contents were designed as research objects. Through thermodynamic calculation, the controlled rolling and controlled cooling processes were simulated by the Gleeble-3800 thermal simulator, and the continuous cooling transformation behavior of the microstructure was analyzed. The microstructure and the second phase of the steel were characterized by scanning electron microscope and transmission electron microscope. Through this study, an attempt was made to accurately reveal the role of Nb content and different cooling rates on the bainite strengthening as well as the morphology and dimensions of composite precipitated phases in steel bars, and to provide some theoretical basis for the composition and heat treatment process design of microalloyed steel bars.

### 1.1. Experimental Materials

Two sets of steels with different Nb contents were designed, and the effect of Nb/Ti/V composite strengthening and thermal deformation on the continuous cooling transformation behavior of High-Strength rebar was analyzed. In order to obtain a high volume fraction of precipitates without reducing the weldability of rebar, the carbon content should be less than 0.25 wt.%. The addition of Si and Mn to High-Strength rebar mainly played a role in solid solution strengthening. The addition of the microalloying elements Nb, V, and Ti mainly played a role in precipitation strengthening and fine grain strengthening.

The raw materials were a 500 MPa grade steel bar supplied by a steel company and processed into a 20 mm diameter and 50 mm high cylindrical bar (Φ20 × 250 mm). The alloys used to adjust the elemental content were purchased from a company, mainly ferrosilicon, ferromanganese, niobium, vanadium, and titanium alloys. The experimental steels were smelted in an intermediate frequency induction melting furnace and cast into a Φ20 × 500 mm steel ingot after melting. The chemical composition of the experimental steels was analyzed by carbon–sulfur analyzer, nitrogen–hydrogen–oxygen analyzer, and inductively coupled plasma atomic emission spectrometer analyzer, as shown in Table 1.

The carbon equivalent C_eq_ (%) can be calculated according to Equation (1).

(1) $$C_{\mathrm{eq}}=C+\frac{\mathrm{Mn}}{6}+\frac{V}{5}$$

### 1.2. Experimental Process

**Figure 1:**
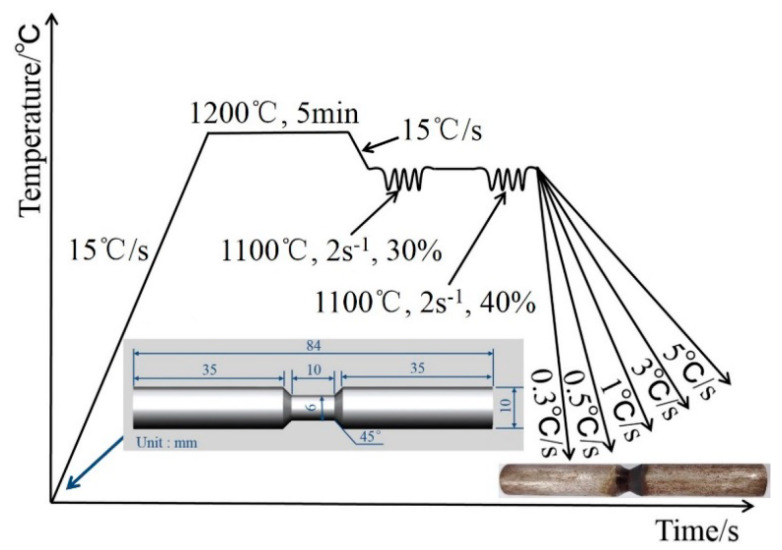
Thermal simulation process of experimental steels and photos of specimens before and after thermal simulation.

The steel ingots were processed into Φ10 × 84 mm dumbbell-shaped specimens by wire cutting, and the Gleeble-3800 thermal simulator (Dynamic Systems Inc., Poestenkill, NY, USA) equipped with a thermal expansion meter was used to conduct a thermal simulation compression experiment. In addition, the thermal expansion curves of the experimental steels at different cooling rates were measured. The thermal simulation process of experimental steels and photos of specimens before and after thermal simulation are given in Figure 1.

The samples were soaked at 1200 °C for 5 min and cooled to 1100 °C at 15 °C/s and carried out the two deformations simulating hot rolling passes, and the deformation amounts were 30% and 40%, respectively. After the final rolling temperature of 1100 °C, the samples cooled at different cooling rates (0.3 °C/s, 0.5 °C/s, 1 °C/s, 3 °C/s, 5 °C/s). In this study, samples were soaked at 1200 °C, which ensured that Nb, Ti, and V were uniformly dissolved in austenite. In the austenite recrystallization temperature range (1100 °C), the samples carried out two deformations simulating hot rolling passes, and the austenite grains were strengthened by repeated recrystallization. Under the conditions of different cooling rates, the different types of microstructural transformation and precipitation behavior were analyzed.

### 1.3. Experimental Methods

The thermodynamic evolution of the phases was performed using the JMatPro (database ver. 7.0) software package to determine the heat treatment process, and the continuous cooling transformation (CCT) of the experimental steel was also calculated. According to the thermal expansion data of different cooling rates recorded by thermal simulation, the thermal expansion curve was drawn by a drawing software (origin 8.5), and then the phase transition temperature under different cooling rates was found by the tangent method, and the phase transition is determined by combining the metallographic structure of the steel. Finally, the deformation continuous cooling transformation (DCCT) diagram of the experimental steel was drawn.

The intermediate deformation zone of the samples after thermal simulation was taken as the research object of the following work. In the middle deformation, the sample size required for the following experiments was processed by wire electrical discharge machining. It is worth noting that the tensile samples and other tested samples were not cut from the same thermal simulation sample.

Samples at different cooling rates were processed into Φ10 × 10 mm metallographic samples. After the samples were coarsely and finely ground and polished, the samples were uniformly corroded with a 4% nitric acid alcohol solution. The OLYMPUSGX71 metallurgical microscope and SUPRA40 scanning electron microscope (SEM) (Carl Zeiss Microscrolmaging GmbH, Jena, Thuringia, Germany) were used to characterize the microstructural characteristics of the samples. The proportion of ferrite and pearlite in the samples was calculated by the Image-Pro Plus (ver. 6.0) software (Media Cybernetics, Inc., Rockville, MD, USA). The Nano Measurer (ver. 1.2) software (Xu Jie, Department of Chemistry, Fudan University, Laboratory of Surface Chemistry and Catalysis, Shanghai, China) was used to measure the ferrite and precipitates grain size.

The Tecnai G2 F30 S-TWIN high-resolution transmission electron microscope (TEM) (FEI, Hillsborough, OR, USA) was used to characterize the precipitation behavior of Nb/Ti/V. The working voltage was 200 kV. The energy dispersive spectroscopy (EDS) equipped with TEM analyzed the composition of Nb/Ti/V. The TEM samples were processed into a Φ5 × 1 mm small disc and ground to a thickness of 0.1 mm. The samples were first mechanically thinned to 30 μm and then performed ion thinning.

**Figure 2:**
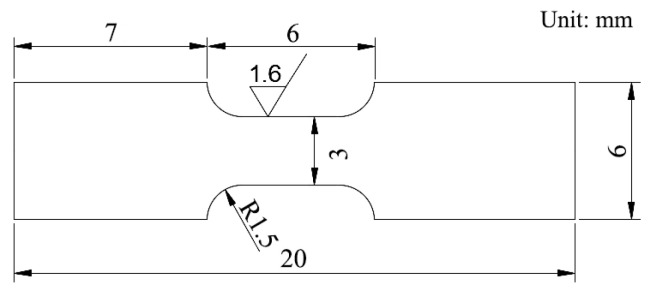
Tensile specimen.

The heat-treated specimens were processed into tensile test standard parts, as shown in Figure 2, where the thickness was 2 mm. The tensile tests were conducted at room temperature on the MTS810 universal tensile testing machine.

## 2. Results and Discussion

### 2.1. Continuous Cooling Transformation and Thermodynamic Analysis

**Figure 3:**
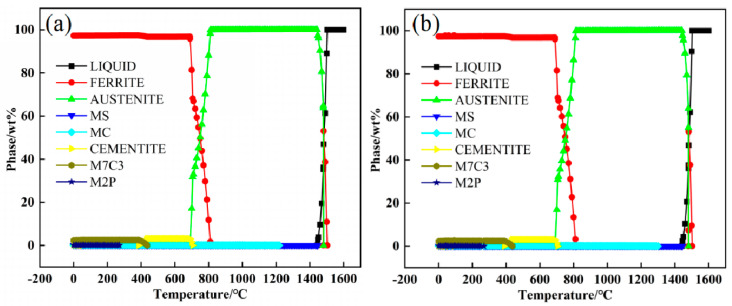
Thermodynamic evolution of the phases determined using JMatPro software: (**a**) steel 1#, (**b**) steel 2#.

The phase thermodynamic evolution of the experimental steels was calculated using JmatPro (database ver. 7.0) software [27,28], and the results are shown in Figure 3. It can be seen from Figure 3a that the liquidus temperature of steel 1# is 1502.7 °C, the solidus temperature is 1442.8 °C, the second phase carbide precipitates at about 1218.7 °C, and the austenite transformation starting temperature (A_c3_) is 811.4 °C. It can be seen from Figure 3b that the liquidus temperature of steel 2# is 1502.4 °C, the solidus temperature is 1440.0 °C, the second phase carbide precipitates at about 1300 °C, and A_c3_ is 813.0 °C. Therefore, when austenitizing at 1200 °C, both groups of steel are almost 100% austenite with a small amount of carbide and sulfide.

**Figure 4:**
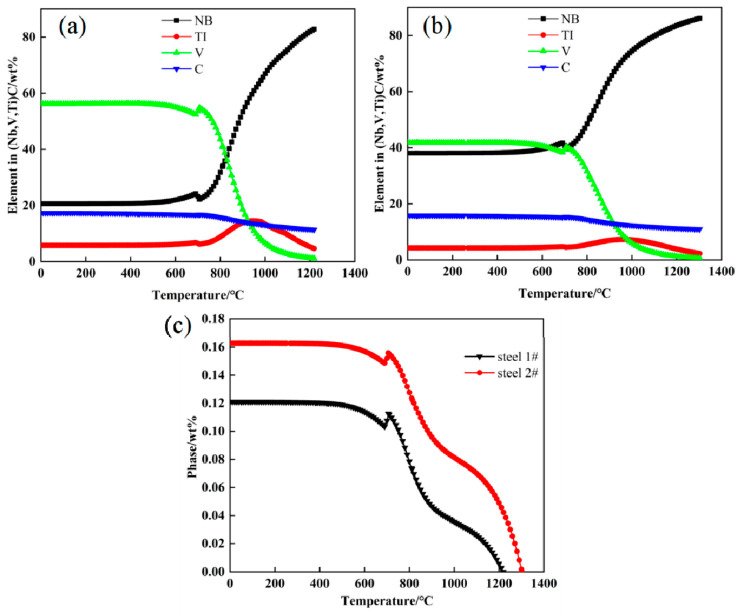
Thermodynamic calculations of precipitation processes using JMatPro software: different elements dissolved in (Nb, V, Ti) C of (**a**) steel 1#, and (**b**) steel 2#; (**c**) precipitation of (Nb, V, Ti)C compound.

The thermodynamics of precipitate precipitation in the experimental steel were calculated using JmatPro software, as shown in Figure 4. It can be seen from Figure 4a that the Nb content in the precipitates of steel 1# decreases with decreasing temperature, while the V content decreases in the opposite direction, and the Ti content first increases and then decreases at high temperature. After the temperature drops to 500 °C, it reaches a relatively stable state when the precipitation is basically completed. At temperatures higher than 870 °C, the precipitation of Nb-rich precipitates mainly. At temperatures below 870 °C, V-rich carbides were precipitated. As can be seen from Figure 4b, steel 2# mainly precipitates as Nb-rich carbides at higher than 813 °C, and mainly precipitates as V and Nb-rich carbides at lower than 813 °C. As can be seen from Figure 4c, the precipitation rate is fastest within 710–920 °C during the transformation of austenite to ferrite. Due to the increase in Nb content, the amount of precipitate in steel 2# has a great elevation from 0.12 to 0.16 wt.%.

**Figure 5:**
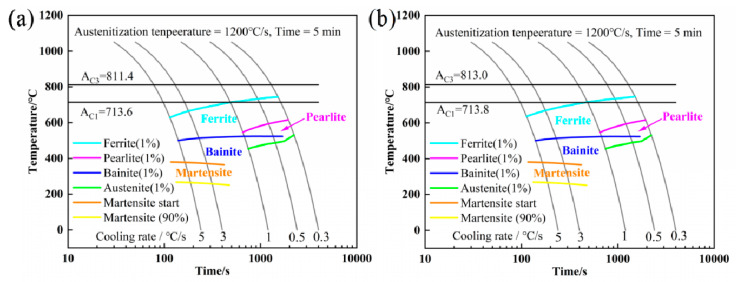
CCT diagram calculated using the JMatPro software: (**a**) steel 1#, and (**b**) steel 2#.

Figure 5 shows the CCT of the experimental steels using JmatPro software. Except for the increase in A_c3_ in steel 2#, there is no significant change in the phase transformation zone of the two groups of steels. Pearlite transformation occurs at a low cooling rate, bainite transformation occurs at medium and high cooling rates, and martensite transformation also occurs at high cooling rates.

**Figure 6:**
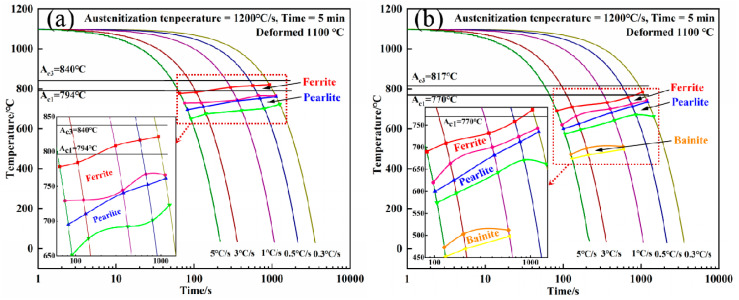
DCCT diagram of (**a**) steel 1# and (**b**) steel 2#.

Figure 6 shows the DCCT diagram based on the thermal expansion curve of the experimental steel measured during the thermal simulation. From Figure 6a, it can be seen that ferrite phase transformation and pearlite phase transformation occurred in underercooled austenite of steel 1#, and the ferrite phase transformation area is larger than the pearlite phase transformation area. From Figure 6b, it can be seen that with the increase in cooling rate, the ferrite phase transformation area in steel 2# has a tendency to increase while the pearlite phase transformation area is gradually reduced. When the cooling rate is greater than 1 °C/s, the bainite phase transformation begins to appear in steel 2#, and the ferrite phase transformation area and the pearlite phase transformation area are larger than the bainite phase transformation area. Comparing the phase transformation temperatures of the two groups of steels, the temperature of A_c3_ and the austenite transformation end temperatures (A_c1_) of steel 2# decreased, the temperature of A_r3_ also decreased, and the ferrite phase transformation temperature area was significantly reduced.

During the continuous rolling process, the density and number of crystal defects such as dislocations and sub-grain boundaries increase, and the Nb/Ti/V composite carbides further precipitate on austenite grain boundaries and crystal defects. Due to the effect of thermal deformation and further precipitation of Nb composite carbides, the stability of supercooled austenite reduces, and the free energy of the system increases, which improves the driving force of austenite phase transformation [29,30]. So thermal deformation causes a significant increase in the phase transformation temperature. On the other hand, when the deformed austenite undergoes recrystallization (at least partial recrystallization), the dislocation density decreases, the grain size refines, and the stability of the austenite after recrystallization is higher, thus prolonging the time required for the transformation to occur [31]. It can be seen from Figure 4 that there are more second-phase carbides precipitated in steel 2# at 1100 °C, which facilitates the further refinement of austenite grains during rolling and further improves austenite stability. Therefore, the phase transition area of steel 2# in Figure 6b shifts downward. When the cooling rate is greater than or equal to 1 °C/s, the transformation of undercooled austenite to bainite is promoted.

### 2.2. Microstructural Transformation

**Figure 7:**
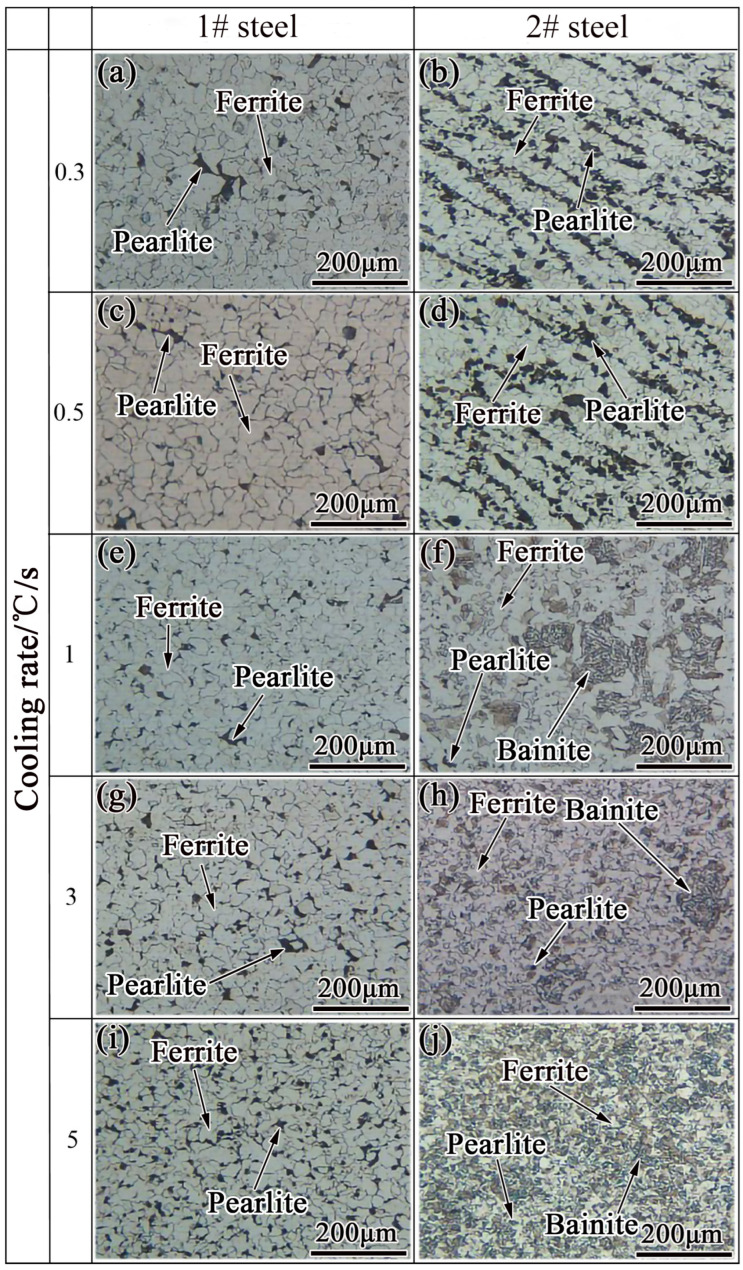
Microstructure transformation of (**a**,**c**,**e**,**g**,**i**) steel 1# and (**b**,**d**,**f**,**h**,**j**) steel 2# at different cooling rates.

The microstructural transformation of steels 1# and 2# under the conditions of different cooling rates is given in Figure 7. At a cooling rate of 0.3–5 °C/s, both steels 1# and 2# undergo ferrite phase transformation and pearlite phase transformation, with ferrite showing a polygonal shape and pearlite being distributed in lamellae on the ferrite matrix. With the increase in cooling rate, the volume fraction of ferrite and pearlite decreases, and the microstructure after the phase transformation significantly refines. The bainite transformation starts in steel 2# at a cooling rate greater than or equal to 1 °C/s, while there was no bainite phase transformation in steel 1#.

**Figure 8:**
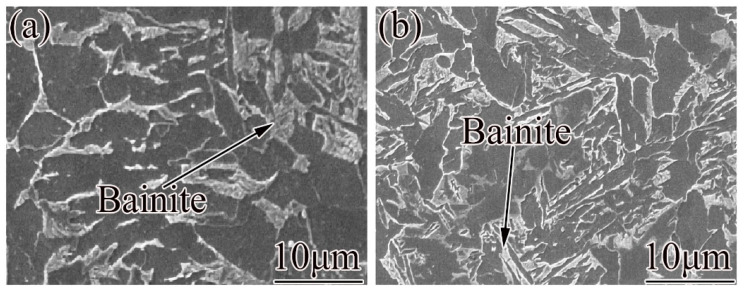
Bainite morphology of steel 2# at different cooling rates: (**a**) 1 °C/s, (**b**) 5 °C/s.

Figure 8 shows SEM images of the bainite morphology of steel 2# at cooling rates of 1 °C/s and 8 °C/s. Figure 8a shows that when the cooling rate is 1 °C/s, the bainite is lath-shaped, consisting of lath ferrite, lamellar cementite, and a small number of carbides. Figure 8b shows the microstructure morphology at a cooling rate of 5 °C/s. The bainite is lamellar and lath. Compared with 1 °C/s, the lath ferrite, lamellar cementite, and carbides become more refined. As the increase of cooling rate, the transformation temperature decreases, the degree of subcooling becomes larger, and the diffusion capacity of carbon atoms is weakened [32]. At this time, the carbon atoms form finer carbides on some crystal faces in ferrite, which makes the ferrite strips finer, the cementite finer and more dispersed, and the bainite laths formed thinner. Therefore, at a high cooling rate, fine lath bainite is easily formed in steel 2#.

### 2.3. Phase Transformation Characteristics

**Figure 9:**
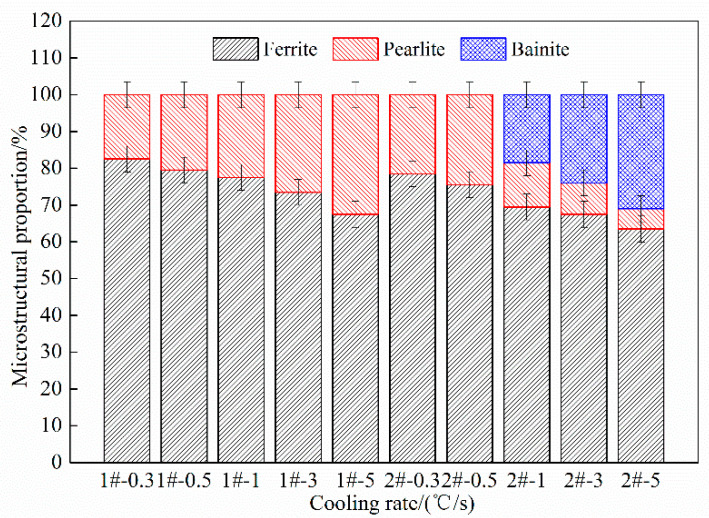
Microstructure proportions of steels 1# and 2# at different cooling rates.

The microstructural proportions of steels 1# and 2# under the conditions of different cooling rates are given in Figure 9. It can be seen from Figure 9 that when the cooling rate increases from 0.3 °C/s to 5 °C/s, the ferrite transformation of steel 1# decreases from 82.5% to 67.5%, while the pearlite transformation increases from 17.5% to 32.5%. For steel 2#, the ferrite transformation decreases from 78.5% to 63.5%, and the pearlite transformation decreases from 21.5% to 5.5%. When it increases from 1 °C/s to 5 °C/s, the bainite transformation increases from 18.5% to 31%.

**Figure 10:**
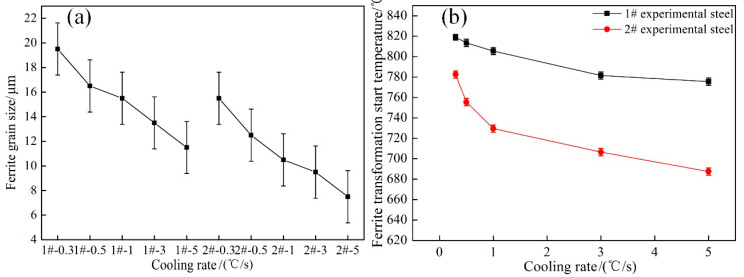
Ferrite grain size (**a**) and ferrite transformation start temperature (**b**) of steels 1# and 2# at different cooling rates.

Ferrite grain size and ferrite transformation start temperature of steels 1# and 2# at different cooling rates are given in Figure 10. From Figure 10a, it can be seen that when the cooling rate is within 0.3–5 °C/s, the ferrite grain size in steel 1# decreases from19.5 ± 2 to 11.5 ± 2 μm, the ferrite grain size in steel 2# decreases from 15.5 ± 2 to 7.5 ± 2 μm. With the increase in cooling rate, the ferrite grain size in steels 1# and 2# is gradually refined, and the ferrite grain size in steel 2# is smaller than that in steel 1#. Figure 10b shows that when the cooling rates are within 0.3–5 °C/s, the ferrite starting phase transformation temperature range is 819–775.5 °C in steel 1# and 782.5–687.5 °C in steel 2#, and the ferrite starting phase transformation temperature decreases with the increase of the cooling rate for both groups of steel.

When the cooling rate is within 0.3–5 °C/s, ferrite and pearlite transformations occur in steels 1# and 2# (as shown in Figure 7), which belong to the diffusion-type phase transformation. The phase transformation process involves the diffusion of carbon, iron, and alloying element atoms. Therefore, the nucleation-growth process of the new phase is closely related to the diffusion rate of atoms. The greater the diffusion of atoms, the faster the diffusion rate. The self-diffusion coefficient of austenite grain boundaries and the self-diffusion coefficient of austenite intragranular in steel are shown in Equations (2) and (3) [33].

(2) $$D_{\mathrm{Grain}\;\mathrm{boundary}}^{\gamma -\mathrm{Fe}}=2.3\exp\left(-\frac{30,600}{RT}\right)$$

(3) $$D_{\mathrm{Intra}-\mathrm{granular}}^{\gamma -\mathrm{Fe}}=0.16\times 10^{-6}\exp\left(-\frac{64,000}{RT}\right)$$

According to Equations (2) and (3), the self-diffusion coefficient of austenite grain boundaries is 10^7^ times that of the austenite intragranular self-diffusion coefficient. This is because the diffusion activation energy of austenite at the grain boundary, phase boundary, dislocation, and sub-grain boundary defects is much lower than that inside austenite grains. The diffusion coefficient of atoms on these defects is greater than the bulk diffusion coefficient inside the grains, and nucleation is more likely to occur. The steels 1# and 2# are continuously compressed by 30% and 40% at 1100 °C, the austenite occurs recrystallization and is repeatedly refined, resulting in a large number of deformation bands, dislocations, sub-grain boundaries, and other defects in austenite, which lead to an increase in the density of defects in austenite, and these defects can not only store a large amount of distortion energy but also facilitate the diffusion of atoms, thereby increasing the nucleation rate of ferrite.

According to the ferrite transformation start temperature of the steel, the increase in cooling rate leads to an increase in supercooling degree, and the increase in niobium content furthers the solid solution of niobium in steel. The mismatch between the niobium and iron matrix is relatively large, and the niobium element is easy to segregate at the grain boundaries. At this time, the phase interface reduces the interface energy, and the solid solution of atoms has a drag effect on the migration of grain boundaries. The niobium element and carbon element have strong interactions and reduce the activity of carbon atoms, inhibiting the diffusion of carbon atoms [34]. Therefore, the increase in Nb and cooling rate reduce the ferrite transformation temperature. With the increase of cooling rate, the A_r3_ decreases obviously, and the driving force of phase transformation increases [35]. According to the relationship between ferrite grain size (*d*) and Ar_3_, as shown in Equation (4) [36]. The results show that the decrease of Ar_3_ also leads to a significant decrease in ferrite grain size after the phase transformation:

(4) $$d={\left[f\cdot \exp\left(B-\frac{E}{{\mathrm{Ar}}_{3}}\right)\right]}^{\frac{1}{3}}$$

where *f* is the ferrite volume fraction, %; *B* is the parameter related to the austenite grain size before the transformation; and *E* is the coefficient, K.

### 2.4. Precipitation Behavior of Nb/Ti/V Composite Strengthening

**Figure 11:**
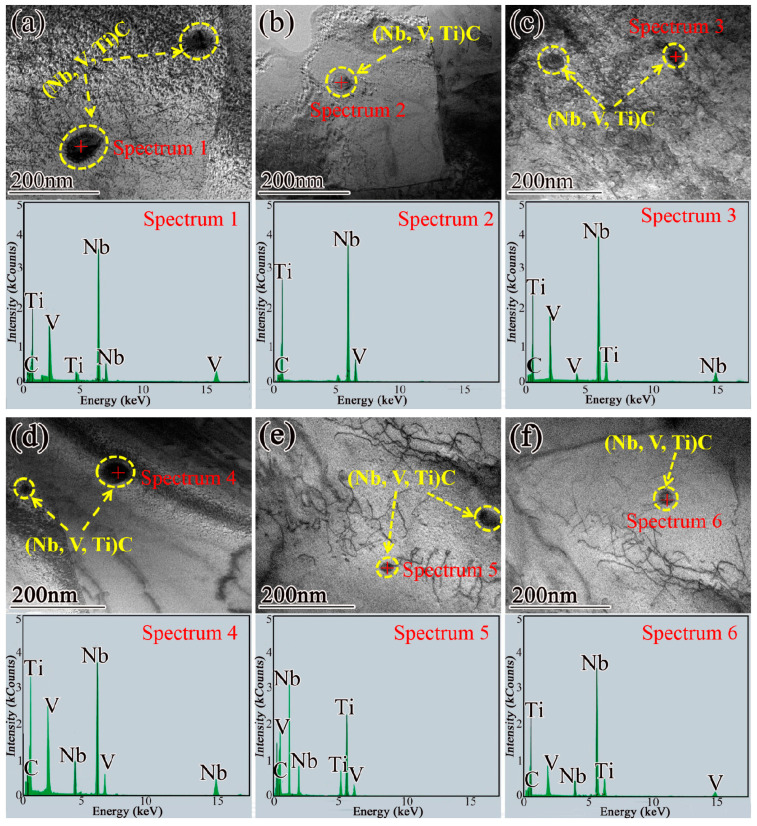
TEM images of precipitates in (**a**–**c**) steel 1# and (**d**–**f**) steel 2# at different cooling rates: (**a**,**d**) 0.3 °C/s, (**b**,**e**) 1 °C/s, (**c**–**f**) 5 °C/s.

The TEM and EDS images of precipitates of the steels 1# and 2# at different cooling rates are given in Figure 11, in which Figure 11a–c are steel 1# at 0.3 °C/s, 1 °C/s, and 5 °C/s, respectively, and Figure 11d–f show steel 2# at 0.3 °C/s, 1 °C/s, and 5 °C/s, respectively. It can be seen from Figure 11 that under different cooling rates, both steels 1# and 2# have (Nb, Ti, V)C precipitation, and the shape is elliptical, evenly distributed on the ferrite matrix or dislocation. The precipitates were measured, and it was found that when the cooling rates were 0.3 °C/s, 1 °C/s, and 5 °C/s, the average particle size of steel 1# was 50 nm (Figure 11a), 30 nm (Figure 11b), and 20 nm (Figure 11c), respectively, while the average particle size of steel 2# was 30 nm (Figure 11d), 20 nm (Figure 11e), and 15 nm (Figure 11f), respectively. Therefore, with the increase in cooling rate, the average particle size of (Nb, Ti, V)C precipitates in steels 1# and 2# decreases, and the precipitate size in steel 2# of 0.062 wt.% Nb is generally smaller. This orientation relationship exists when precipitates (Nb, Ti, V)C form precipitates at the γ/α interface or in the transformed α matrix during the γ→α transformation [37,38,39]. Therefore, in High-Strength rebar, the precipitates (Nb, Ti, V)C not only exist in the form of strain-nduced precipitation in the deformed γ but also form in the process of γ transformation and in the interior of α after transformation.

At low cooling rates (0.3 °C/s and 0.5 °C/s), the Nb/Ti/V precipitates may first nucleate at austenite grain boundaries and dislocation lines before the ferrite transformation. Its nucleation function mainly has the following two aspects: First, the preferentially nucleated Nb/Ti/V precipitates consume the distortion energy at the crystal defect boundaries such as sub-grain boundaries and deformation zones and have less influence on the distortion energy at the intra-granular defect, resulting in the increase in driving force of the ferrite transformation, which is not conducive to the ferrite transformation. Second, the preferential precipitation of Nb/Ti/V precipitates reduces the solid solution content of carbon, niobium, titanium, and vanadium in the deformed austenite; the reduction in solid solution content of niobium weakens its solute drag effect and results in the result in the ferrite nucleation rate. Therefore, based on the above analysis, the effect of the second aspect is more obvious, namely, the precipitation of Nb/Ti/V precipitates in the deformed austenite promotes the ferrite transformation.

**Figure 12:**
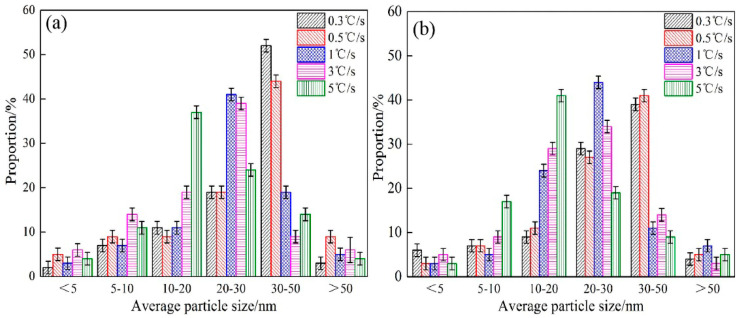
Size distribution of precipitates in (**a**) steel 1# and (**b**) steel 2# at different cooling rates.

Figure 12 shows the size distribution of Nb/Ti/V precipitates in steels 1# and 2# at different cooling rates. From Figure 12, it can be seen that at a cooling rate less than or equal to 0.5 °C/s, the maximum percentage of particle size of Nb/Ti/V precipitates for both steels is 30–50 nm, while the percentage of particle size of steel 2# in the range of 20–30 nm is significantly increased. When the cooling rate is increased to 1 °C/s or 3 °C/s, the largest proportion of precipitate size is between 20 and 30 nm, while the percentage of precipitates smaller than 20 nm is significantly higher for steel 2# than for steel 1#. When the cooling rate is increased to 5 °C/s, the particle size of precipitates is concentrated in the range of 10–20 nm, and the proportion of precipitates with a size less than 10 nm increases significantly. It can be seen that with the increase in cooling rate and Nb content in steel, the particle size of (Nb, Ti, V)C precipitates obviously decreases.

Combining the above precipitation behavior analysis with the principle of the Nb element, when the cooling rate increases, the decrease of temperature is accelerated, and the atomic diffusion rate decreases sharply with the decrease of temperature, so the growth rate of precipitated phase nuclei decreases. According to the thermodynamic calculation of (Nb, Ti, V)C precipitates in Figure 4, steel 2# mainly precipitates Nb-rich or Nb and V-rich carbides. Nb precipitates preferentially at high temperatures, and the precipitation rate is faster. Therefore, the particle size of Nb/Ti/V precipitates can be effectively reduced by appropriately increasing the cooling rate and the content of Nb elements in the steel.

### 2.5. Mechanical Properties

**Figure 13:**
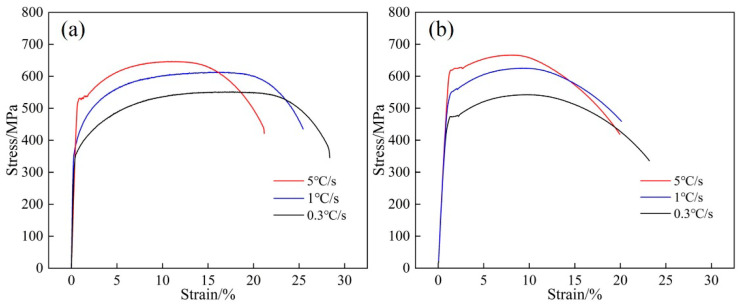
The engineering stress–strain curves for (**a**) steel 1# and (**b**) steel 2# at different cooling rates.

The yield strength (YS), tensile strength (TS), and total elongation (TE) of steels 1# and 2# at cooling rates of 0.3 °C/s, 1 °C/s, and 5 °C/s are listed in Table 2, and its engineering stress-strain curve is exhibited in Figure 13. It shows that the YS and TS increase significantly with the increase in cooling rate in both steels 1# and 2#, however, the TE showed a slight decrease. As the Nb content increased from 0.025 to 0.062 wt.%, the strength of steel has been greatly improved, but the product of tensile strength and elongation (PSE) decreased. According to the previous research on the microstructure and precipitates in the experimental steel, increasing Nb content has a good improvement on the microstructure and precipitates. At the same time, it also significantly improves the strength of steel, and a small reduction in the comprehensive performance of steel is acceptable. In addition, the comprehensive performance of steel 2# is greatly improved when the cooling rate is 5 °C/s. According to the microstructure statistics of the steels in Figure 9, the bainite of steel 2# increases rapidly to 31% at 5 °C/s, which indicates that the increase of bainite in the experimental steel has a great influence on its comprehensive properties.

## 3. Conclusions

In this study, under the conditions of thermal deformation and different cooling rates, the effect of Nb content and Nb/Ti/V composite strengthening on the continuous cooling transformation behavior of High-Strength rebar was discussed. The main conclusions were as follows:

1. Ferrite transformation and pearlite transformation occurred in the experimental steels. The bainite transformation started to occur in the experimental steel when the Nb content increased from 0.025 wt.% to 0.062 wt.% and the cooling rate was greater than or equal to 1 °C/s, and the volume fraction of bainite increased with the increase in the cooling rate.
2. By controlling the thermal deformation and subsequent cooling rate, a ferrite structure with finer grains, and large number can be obtained. With the increase in cooling rate and Nb content, the volume fraction of ferrite and grain size in the experimental steel decreased significantly; the ferritie grain size was reduced from 19.5 to 7.5 μm.
3. The experimental steels mainly precipitated Nb-rich carbides in the austenite phase at high temperature, and the precipitation rate was the fastest during the transformation from the austenite to ferrite phase. During and after the complete transformation from austenite to ferrite, V-rich carbides were mainly precipitated in steel 1# and Nb and V-rich carbides were mainly precipitated in steel 2#. The main precipitates observed in the experimental steel were (Nb, Ti, V)C, which were mainly distributed in the ferrite matrix and dislocation lines. Its morphology was roughly elliptical, and its average particle size was between 10 and 50 nm. The particle size of (Nb, Ti, V)C precipitates can be effectively reduced by appropriately increasing the cooling rate or the Nb content in the steel.
4. When the Nb content was increased to 0.062 wt.% or the cooling rate was increased, the yield strength and tensile strength could be increased to 618.9 MPa and 666.5 MPa, respectively, but the elongation of the steel was reduced. However, the comprehensive mechanical properties of steel 2# have been significantly improved at a cooling rate of 5 °C/s.

## Figures and Tables

**Table 1:** Chemical composition of the steels (mass fraction, wt.%).

No.	C	Si	Mn	P	S	Nb	V	Ti	C_eq_
1#	0.23	0.45	1.55	0.019	0.013	0.025	0.068	0.007	0.50
2#	0.23	0.46	1.54	0.018	0.012	0.062	0.065	0.007	0.50

**Table 2:** Mechanical properties of steels.

Steels	Cooling Rate/°C/s	YS/MPa (Error ± 2%)	TS/MPa (Error ± 2%)	TS/YS	TE/% (Error ± 2%)	PSE/%
1#	0.3	363.5	551.8	1.52	28.4	156.7112
	1	391.5	613.2	1.57	25.4	155.7528
	5	531.6	646.5	1.22	21.2	137.058
2#	0.3	474.5	542.3	1.14	23.2	125.8136
	1	551.9	625.2	1.13	20.1	125.6652
	5	618.9	666.5	1.08	19.9	132.6335

## Data Availability

The data used to support the findings of this study are included in the article.
